# Supplementary figures and images for: Morphological variability within the indigenous sheep population of Benin
Source: PLoS One. 2021 Oct 19;16(10):e0258761. doi: 10.1371/journal.pone.0258761 (PMC8525752; doi:10.1371/journal.pone.0258761)

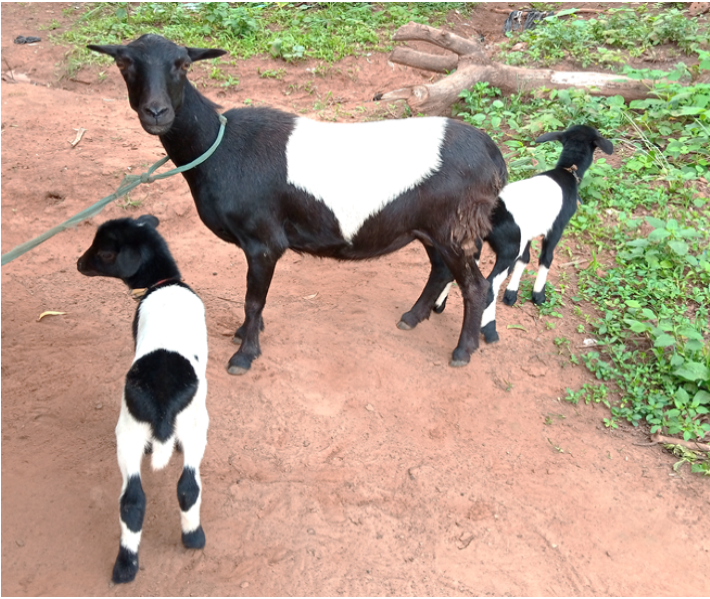

Supplement: S1 Fig — (TIFF) [file pone.0258761.s001.tiff]

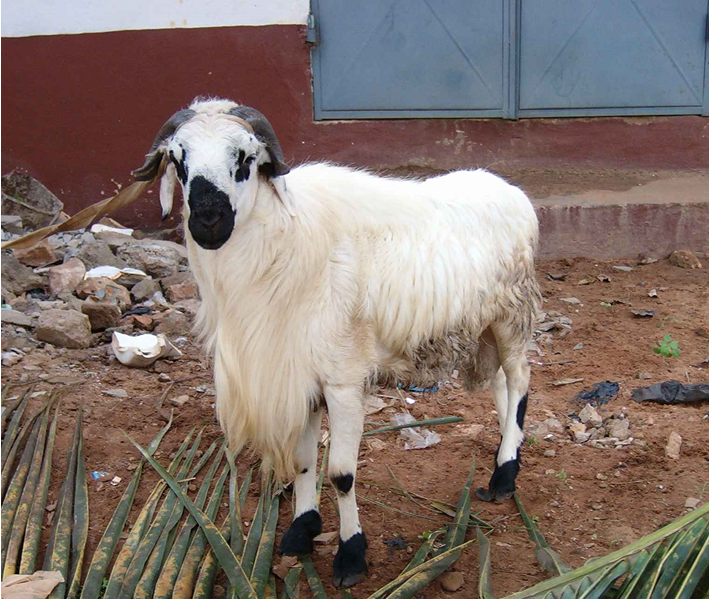

Supplement: S2 Fig — (TIFF) [file pone.0258761.s002.tiff]

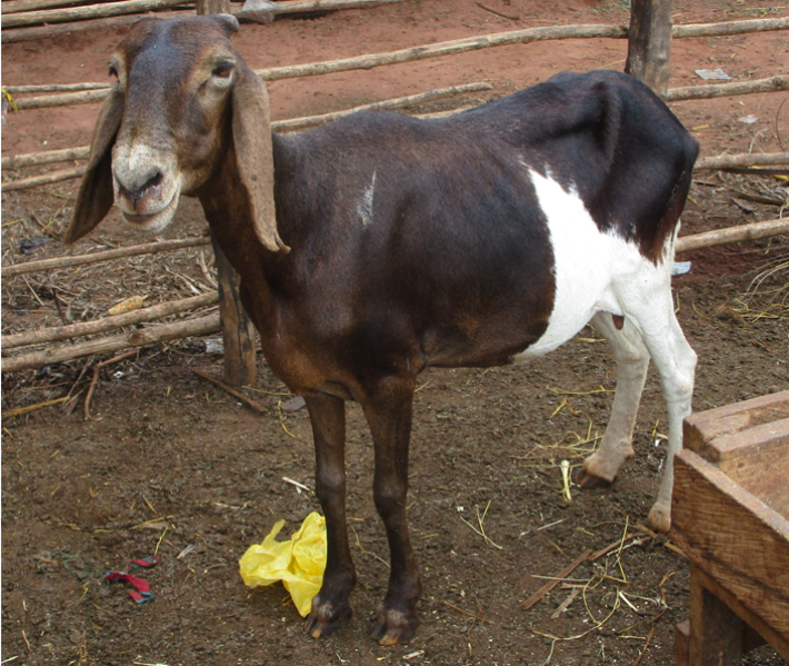

Supplement: S3 Fig — (TIF) [file pone.0258761.s003.tif]

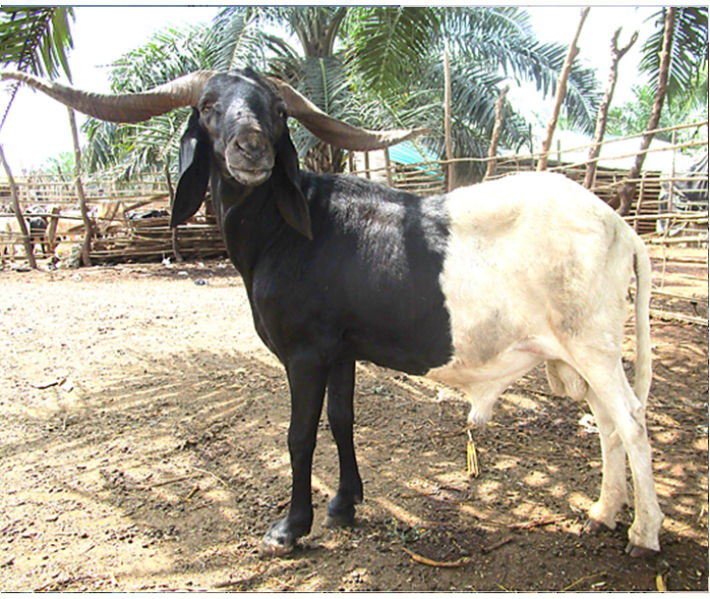

Supplement: S4 Fig — (TIFF) [file pone.0258761.s004.tiff]

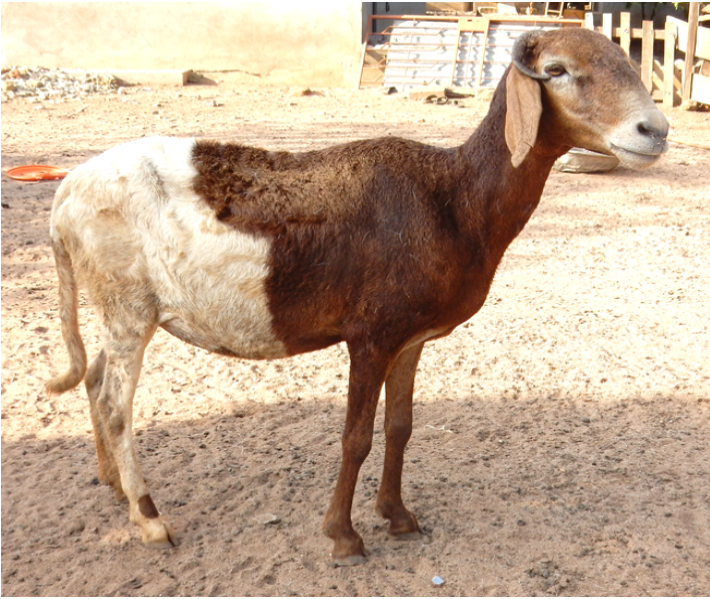

Supplement: S5 Fig — (TIFF) [file pone.0258761.s005.tiff]

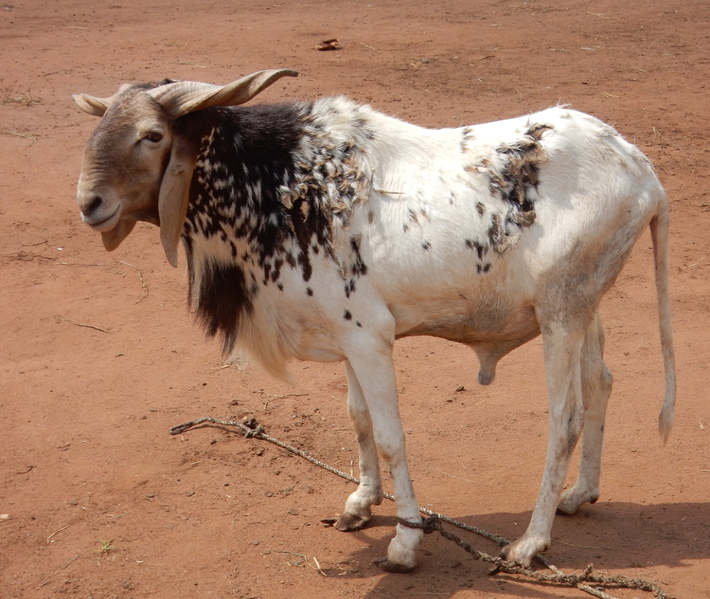

Supplement: S6 Fig — (TIFF) [file pone.0258761.s006.tiff]
