# Supplementary material for: Morphological variability within the indigenous sheep population of Benin
Source: PLoS One. 2021 Oct 19;16(10):e0258761. doi: 10.1371/journal.pone.0258761 (PMC8525752; doi:10.1371/journal.pone.0258761)
Supplement: S1 Table — (PDF) [file pone.0258761.s007.pdf]

S1 Table. Least squares means (LSmeans) and standard errors (SEs) and coefficients of variation (CVs) of morphological measurements (cm) across phytogeographic zones

Variables	BNZ		BSZ		BZ		CAZ		MPZ		PlZ		PoZ		VOZ		CZ		ZZ		Overall	
	n=124		n=112		n=167		n=148		n=128		n=110		n=96		n=103		n=105		n=147		(n=1240)	
	LSmean±SE	CV	LSmean±SE	CV	LSmean±SE	CV	LSmean±SE	CV	LSmean±SE	CV	LSmean±SE	CV	LSmean±SE	CV	LSmean±SE	CV	LSmean±SE	CV	LSmean±SE	CV	LSmean±SE	CV
WH	61.86±0.639^{b}	11.51	60.14±0.547^{c}	9.62	58.66±0.419^{d}	9.22	61.56±0.549^{b}	10.86	68.35±0.473^{a}	7.83	54.41±0.507^{f}	9.78	49.20±0.344^{g}	6.85	49.41±0.336^{g}	6.89	50.77±0.39^{g}	7.86	55.89±0.377^{e}	8.17	57.58±5.338	9.27
RH	62.17±0.635^{b}	11.38	60.61±0.564^{b}	9.85	61.35±0.422^{b}	8.90	61.71±0.551^{b}	10.87	68.79±0.474^{a}	7.79	55.16±0.549^{c}	10.44	52.02±0.347^{e}	6.53	50.89±0.312^{e}	6.21	53.67±0.373^{d}	7.13	55.86±0.377^{c}	8.18	58.73±5.381	9.16
SH	38.42±0.518^{c}	15.01	39.79±0.364^{b}	9.68	33.21±0.361^{e}	14.03	39.60±0.395^{b}	12.12	46.00±0.353^{a}	8.68	34.77±0.385^{d}	11.61	25.95±0.261^{g}	9.86	33.68±0.293^{e}	8.84	27.10±0.290^{f}	10.97	35.79±0.305^{d}	10.35	35.81±4.130	11.53
BH	60.36±0.611^{b}	11.28	58.55±0.537^{c}	9.70	59.60±0.418^{bc}	9.06	59.94±0.541^{bc}	10.98	66.80±0.449^{a}	7.60	53.47±0.496^{d}	9.72	50.40±0.342^{f}	6.65	49.37±0.323^{f}	6.63	52.00±0.366^{e}	7.20	54.35±0.368^{d}	8.20	56.99±5.199	9.12
CD	26.89±0.248^{c}	10.25	27.57±0.217^{b}	8.31	26.63±0.161^{c}	7.82	26.74±0.213^{c}	9.69	29.04±0.214^{a}	8.32	24.12±0.228^{e}	9.90	24.01±0.157^{e}	6.40	23.01±0.202^{f}	8.91	24.39±0.136^{e}	5.70	25.46±0.147^{d}	7.01	25.95±2.191	8.45
RD	29±0.298^{a}	11.43	27.75±0.296^{b}	11.30	27.36±0.196^{b}	9.28	28.01±0.257^{b}	11.16	29.45±0.256^{a}	9.83	25.34±0.241^{d}	9.99	24.39±0.187^{e}	7.50	23.05±0.240^{f}	10.56	24.89±0.174^{de}	7.15	26.10±0.199^{c}	9.25	26.73±2.677	10.02
CW	14.6±0.125^{b}	9.52	14.38±0.123^{b}	9.04	15.40±0.083^{a}	6.99	14.53±0.156^{b}	13.05	13.97±0.143^{c}	11.56	13.28±0.114^{de}	9.04	14.51±0.085^{b}	5.72	13.51±0.157^{d}	11.75	14.29±0.096^{bc}	6.86	13.04±0.101^{e}	9.42	14.19±1.360	9.59
SIL	56.19±0.475^{de}	9.42	57.84±0.404^{c}	7.40	59.87±0.329^{b}	7.11	56.89±0.416^{cd}	8.89	63.16±0.421^{a}	7.54	51.75±0.426^{fg}	8.63	55.02±0.346^{e}	6.17	50.89±0.423^{g}	8.43	54.96±0.359^{e}	6.70	52.78±0.280^{f}	6.44	56.20±4.358	7.75
BL	81.77±0746^{c}	10.16	82.37±0.581^{c}	7.47	86.19±0.479^{b}	7.18	82.39±0.575^{c}	8.49	91.25±0.625^{a}	7.74	75.27±0.634^{e}	8.84	77.64±0.513^{d}	6.47	69.31±0.610^{f}	8.92	76.04±0.493^{de}	6.64	76.16±0.444^{de}	7.07	80.39±6.413	7.98
HL	20.2±0.209^{c}	11.52	19.94±0.180^{c}	9.57	16.29±0.116^{e}	9.23	20.84±0.166^{b}	9.70	22.26±0.168^{a}	8.53	18.98±0.160^{d}	8.82	15.07±0.102^{g}	6.66	16.23±0.181^{f}	11.32	15.74±0.107^{e}	6.97	18.83±0.095^{d}	6.13	18.58±1.704	9.18
HW	9.93±0.101^{b}	11.29	9.4±0.076^{c}	8.68	9.80±0.059^{b}	7.82	9.69±0.087^{b}	10.92	10.41±0.101^{a}	11.02	9.38±0.075^{c}	8.36	9.14±0.061^{c}	6.57	8.10±0.069^{e}	8.68	9.14±0.062^{c}	6.96	8.42±0.062^{d}	8.92	9.36±0.870	9.29
EL	13.55±0.356^{b}	29.26	11.96±0.204^{c}	18.02	10.92±0.144^{d}	17.03	13.26±0.263^{b}	24.17	17.70±0.277^{a}	17.71	10.93±0.162^{d}	15.50	8.84±0.104^{f}	11.48	9.91±0.099^{e}	10.16	9.56±0.104^{e}	11.13	10.74±0.154^{d}	17.43	11.87±2.373	19.98
MD	20.03±0.167^{b}	9.30	20.02±0.165^{b}	8.72	18.28±0.096^{d}	6.82	20.25±0.162^{b}	9.76	20.95±0.168^{a}	9.08	18.54±0.129^{cd}	7.28	16.85±0.095^{f}	5.50	18.84±0.159^{c}	8.59	17.70±0.099^{e}	5.73	18.58±0.092^{cd}	6.03	19.07±1.534	8.04
NL	24.02±0.228^{b}	10.55	24.10±0.233^{b}	10.25	21.60±0.169^{d}	10.09	24.24±0.208^{b}	10.46	28.02±0.239^{a}	9.65	21.90±0.227^{d}	10.86	18.68±0.194^{f}	10.15	20.02±0.250^{e}	12.68	20.21±0.234^{e}	11.87	23.20±0.161^{c}	8.39	22.79±2.368	10.39
NG	25.55±0.235^{a}	10.24	23.8±0.204^{b}	9.06	24.01±0.148^{b}	7.94	24.92±0.238^{a}	11.62	24.89±0.236^{a}	10.71	22.72±0.176^{c}	8.11	22.51±0.176^{c}	7.67	20.82±0.215^{d}	10.48	22.41±0.183^{c}	8.39	22.07±0.134^{c}	7.36	23.48±2.207	9.4
TL	31.43±0.687^{b}	24.33	31.00±0.500^{b}	17.09	30.20±0.435^{b}	18.60	31.45±0.527^{b}	20.38	40.65±0.557^{a}	15.51	23.98±0.463^{c}	20.24	22.32±0.301^{d}	13.19	19.00±0.378^{e}	20.17	24.53±0.325^{c}	13.59	23.99±0.355^{c}	17.95	28.31±5.364	18.94
HG	69.81±0.645^{b}	10.30	68.03±0.517^{b}	8.05	68.91±0.393^{b}	7.37	68.72±0.591^{b}	10.46	72.81±0.560^{a}	8.71	62.16±0.539^{c}	9.09	62.41±0.440^{c}	6.91	59.00±0.477^{d}	8.20	63.21±0.388^{c}	6.29	61.52±0.343^{c}	6.76	66.01±5.592	8.47
CC	84.62±0.743^{a}	9.78	77.31±0.778^{c}	10.65	80.82±0.500^{b}	7.99	81.23±0.750^{b}	11.23	82.31±0.664^{b}	9.13	75.11±0.679^{c}	9.48	75.29±0.606^{c}	7.89	71.73±0.760^{d}	10.76	77.45±0.534^{c}	7.07	72.09±0.444^{d}	7.47	78.08±7.247	9.28
AG	78.75±0.762^{a}	10.78	73.25±0.674^{c}	9.74	73.91±0.488^{bc}	8.52	75.56±0.711^{b}	11.44	78.72±0.661^{a}	9.50	69.12±0.656^{d}	9.95	66.75±0.533^{e}	7.83	63.90±0.772^{f}	12.26	69.74±0.503^{d}	7.40	67.15±0.425^{e}	7.67	72.06±6.976	9.68
BD	36.74±0.405^{b}	12.26	33.82±0.392^{c}	12.27	33.26±0.268^{c}	10.43	36.27±0.426^{b}	14.29	38.67±0.393^{a}	11.49	31.13±0.313^{d}	10.53	28.41±0.260^{f}	8.95	29.87±0.264^{e}	8.98	29.95±0.259^{e}	8.86	31.34±0.267^{d}	10.35	33.22±3.788	11.4
RL	13.23±0.169^{b}	14.25	11.94±0.158^{c}	13.97	13.39±0.118^{b}	11.35	13.25±0.146^{b}	13.39	13.72±0.135^{b}	11.16	11.84±0.158^{c}	14.00	13.21±0.152^{b}	11.23	11.71±0.144^{c}	12.45	14.30±0.167^{a}	11.99	11.23±0.124^{d}	13.43	12.79±1.626	12.71
RW	10.48±0.146^{d}	15.51	10.26±0.175^{d}	18.09	15.49±0.092^{a}	7.67	9.77±0.089^{ef}	11.10	10.08±0.117^{de}	13.16	9.66±0.117^{f}	12.70	13.57±0.177^{b}	12.80	7.98±0.093^{h}	11.89	13.08±0.149^{c}	11.69	9.02±0.102^{g}	13.67	11.01±1.381	12.54
TC	6.46±0.061^{c}	10.44	6.74±0.056^{b}	8.77	6.73±0.036^{b}	6.88	6.56±0.049^{c}	9.08	7.51±0.073^{a}	11.00	6.05±0.051^{d}	8.86	6.04±0.040^{d}	6.57	5.95±0.053^{d}	9.12	6.00±0.040^{d}	6.80	6.11±0.041^{d}	8.23	6.45±0.570	8.84
HC	17.16±0.138^{d}	8.94	17.11±0.122^{d}	7.56	17.36±0.115^{cd}	8.55	17.04±0.132^{d}	9.42	17.70±0.143^{bc}	9.13	16.14±0.147^{e}	9.53	17.57±0.143^{bcd}	7.99	18.23±0.224^{a}	12.49	18.03±0.165^{ab}	9.38	15.41±0.103^{f}	8.10	17.12±1.574	9.2
BW	28.90±0.717^{b}	27.64	27.21±0.560^{bc}	21.80	25.84±0.414^{c}	20.70	28.41±0.724^{b}	31.02	34.81±0.896^{a}	29.11	21.62±0.476^{d}	23.09	20.43±0.338^{d}	16.21	15.89±0.342^{e}	21.87	21.39±0.318^{d}	15.25	21.14±0.304^{d}	17.41	24.95±6.313	25.31

^{abcdefgh} LSmeans with different letters in rows are significantly different at P≤0.001; SNK's multiple mean comparison test;
MPZ, Mekrou-Pendjari zone; CAZ, Chaîne Atacora zone; BNZ, Borgou-Nord zone; BSZ, Borgou-Sud zone; BZ, Bassila zone; CZ, Coastal zone; PoZ, Pobe zone; PlZ, Plateau zone; VOZ, Oueme Valley zone; ZZ, Zou zone. WH, withers height; RH, rump height; SH, sternum height; BH, back height; CD, chest depth; RD, rump depth; CW, chest width; SIL, scapulo-ischial length; BL, body length; HL, head length; HW, head width; EL, ear length; MD, muzzle diameter; NL, neck length; NG, neck girth; TL, tail length; HG, heart girth; CC, chest circumference; AG, abdominal girth; BD, biscostal diameter; RW, rump width; RL, rump length; TC, cannon bone circumference; BW, body weight.
