# Supplementary material for: Morphological variability within the indigenous sheep population of Benin
Source: PLoS One. 2021 Oct 19;16(10):e0258761. doi: 10.1371/journal.pone.0258761 (PMC8525752; doi:10.1371/journal.pone.0258761)
Supplement: S2 Table — (PDF) [file pone.0258761.s008.pdf]

## S2 Table. Least squares means (LSmeans) and standard errors (SEs) and coefficients of variation (CVs) of morphological indices across phytogeographic zones

| Variables | BNZ | | BSZ | | BZ | | CAZ | | MPZ | | PlZ | | PoZ | | VOZ | | CZ | | ZZ | | Overall | |
|---|---|---|---|---|---|---|---|---|---|---|---|---|---|---|---|---|---|---|---|---|---|---|
| | n=124 | | n=112 | | n=167 | | n=148 | | n=128 | | n=110 | | n=96 | | n=103 | | n=105 | | n=147 | | (n=1240) | |
| | LSmean±SE | CV | LSmean±SE | CV | LSmean±SE | CV | LSmean±SE | CV | LSmean±SE | CV | LSmean±SE | CV | LSmean±SE | CV | LSmean±SE | CV | LSmean±SE | CV | LSmean±SE | CV | LSmean±SE | CV |
| MI | 46.16±0.716$^{b}$ | 17.27 | 44.97±0.655$^{b}$ | 15.40 | 43.81±0.492$^{bc}$ | 14.51 | 45.48±0.770$^{b}$ | 20.61 | 50.65±1.167$^{a}$ | 26.07 | 39.48±0.604$^{e}$ | 16.05 | 41.48±0.583$^{d}$ | 13.76 | 32.13±0.621$^{f}$ | 19.60 | 42.07±0.496$^{cd}$ | 12.08 | 37.71±0.364$^{e}$ | 11.69 | 42.65±7.649 | 17.93 |
| IGS | 1.3±0.011$^{b}$ | 9.20 | 1.18±0.012$^{de}$ | 10.94 | 1.20±0.011$^{d}$ | 11.27 | 1.31±0.014$^{b}$ | 13.30 | 1.36±0.012$^{a}$ | 9.86 | 1.26±0.016$^{c}$ | 13.37 | 1.05±0.012$^{f}$ | 11.29 | 1.16±0.01$^{e}$ | 13.86 | 1.08±0.013$^{f}$ | 12.21 | 1.20±0.010$^{de}$ | 10.05 | 1.22±0.141 | 11.56 |
| IAT | 0.5±0.009$^{b}$ | 19.88 | 0.43±0.005$^{d}$ | 12.73 | 0.41±0.004$^{ef}$ | 13.19 | 0.49±0.007$^{b}$ | 17.59 | 0.61±0.009$^{a}$ | 16.33 | 0.45±0.006$^{c}$ | 12.94 | 0.37±0.004$^{g}$ | 9.44 | 0.43±0.005$^{d}$ | 12.31 | 0.39±0.004$^{f}$ | 9.57 | 0.42±0.005$^{de}$ | 14.25 | 0.45±0.069 | 15.19 |
| USI | 56.42±0.205$^{b}$ | 4.04 | 54.02±0.265$^{cd}$ | 5.19 | 54.46±0.221$^{c}$ | 5.23 | 56.39±0.277$^{b}$ | 5.97 | 57.46±0.213$^{a}$ | 4.20 | 55.55±0.326$^{b}$ | 6.15 | 51.10±0.287$^{e}$ | 5.51 | 53.39±0.320$^{d}$ | 6.08 | 51.81±0.296$^{e}$ | 5.86 | 54.35±0.206$^{c}$ | 4.60 | 54.67±2.88 | 5.28 |
| BI | 9.28±0.051$^{g}$ | 6.12 | 9.92±0.071$^{bcd}$ | 7.57 | 9.78±0.045$^{cde}$ | 5.97 | 9.57±0.058$^{ef}$ | 7.31 | 10.35±0.094$^{a}$ | 10.31 | 9.76±0.065$^{cde}$ | 7.00 | 9.69±0.059$^{ef}$ | 5.93 | 10.10±0.079$^{b}$ | 7.97 | 9.50±0.050$^{f}$ | 5.36 | 9.94±0.05$^{bc}$ | 6.04 | 9.79±0.700 | 7.15 |
| IP | 79.5±0.775$^{e}$ | 10.86 | 86.10±1.061$^{d}$ | 13.04 | 116.70±0.896$^{a}$ | 9.93 | 74.05±0.417$^{f}$ | 6.85 | 73.87±0.792$^{f}$ | 12.13 | 81.91±0.580$^{e}$ | 7.43 | 104.46±2.012$^{b}$ | 18.87 | 69.27±1.210$^{g}$ | 17.72 | 93.30±1.866$^{c}$ | 20.49 | 80.67±0.632$^{e}$ | 9.50 | 86.48±11.49 | 13.29 |
| CDI | 43.58±0.205 $^{d}$ | 5.23 | 45.98±0.265$^{bc}$ | 6.10 | 45.54±0.221$^{c}$ | 6.26 | 43.61±0.277$^{d}$ | 7.72 | 42.54±0.213$^{e}$ | 5.67 | 44.45±326$^{d}$ | 7.69 | 48.90±0.287$^{a}$ | 5.76 | 46.61±0.320$^{b}$ | 6.96 | 48.19±0.296$^{a}$ | 6.30 | 45.65±0.206$^{c}$ | 5.48 | 45.33±2.885 | 6.36 |
| SI | 110.04±0.556 $^{a}$ | 5.63 | 103.93±0.507$^{d}$ | 5.16 | 98.01±0.479$^{e}$ | 6.32 | 108.19±0.514$^{b}$ | 5.78 | 108.33±0.488$^{b}$ | 5.10 | 105.27±0.69$^{cd}$ | 6.87 | 89.53±0.531$^{g}$ | 5.81 | 97.46±0.728$^{e}$ | 7.58 | 92.47±0.528$^{f}$ | 5.85 | 105.87±0.345$^{c}$ | 3.95 | 102.43±5.936 | 5.79 |
| Ba | 0.35±0.006 $^{cd}$ | 19.06 | 0.31±0.008$^{e}$ | 27.74 | 0.51±0.004$^{b}$ | 11.21 | 0.34±0.004$^{d}$ | 15.15 | 0.34±0.006$^{cd}$ | 18.30 | 0.36±0.006$^{c}$ | 17.74 | 0.51±0.007$^{b}$ | 12.78 | 0.30±0.006$^{e}$ | 18.72 | 0.54±0.006$^{a}$ | 12.18 | 0.31±0.007$^{e}$ | 26.5 | 0.39±0.067 | 17.19 |
| IBR | 80.67±0.385$^{e}$ | 5.31 | 85.14±0.363$^{c}$ | 4.52 | 87.03±0.375$^{ab}$ | 5.57 | 83±0.338$^{d}$ | 4.96 | 86.94±0.387$^{ab}$ | 5.04 | 83.4±0.383$^{d}$ | 4.81 | 88.35±0.526$^{a}$ | 5.83 | 86.53±0.715$^{bc}$ | 8.39 | 87.03±0.423$^{ab}$ | 4.98 | 85.90±0.314$^{bc}$ | 4.43 | 85.34±4.636 | 5.43 |
| PI | 1.32±0.007 $^{c}$ | 6.33 | 1.26±0.003$^{e}$ | 2.91 | 1.43±0.006$^{b}$ | 5.55 | 1.28±0.002$^{d}$ | 2.25 | 1.25±0.002$^{e}$ | 2.02 | 1.3±0.006$^{d}$ | 4.54 | 1.51±0.008$^{a}$ | 4.92 | 1.26±0.007$^{e}$ | 5.81 | 1.50±0.009$^{a}$ | 6.30 | 1.28±0.004$^{d}$ | 3.86 | 1.34±0.064 | 4.77 |
| IC | 49.37±0.409 $^{c}$ | 9.22 | 46.51±0.335$^{d}$ | 7.62 | 60.37±0.335$^{a}$ | 7.16 | 46.54±0.244$^{d}$ | 6.38 | 46.88±0.387$^{d}$ | 9.33 | 49.59±0.352$^{c}$ | 7.44 | 60.78±0.384$^{a}$ | 6.18 | 50.55±0.731$^{c}$ | 14.69 | 58.20±0.362$^{b}$ | 6.37 | 44.75±0.294$^{e}$ | 7.97 | 51.20±4.278 | 8.35 |
| BR | 1±0.001 $^{a}$ | 1.43 | 0.99±0.002 $^{ab}$ | 1.99 | 0.96±0.002 $^{d}$ | 2.08 | 1±0.001 $^{a}$ | 0.81 | 0.99±0.002 $^{ab}$ | 1.80 | 0.99±0.004 $^{b}$ | 4.01 | 0.95±0.002 $^{e}$ | 2.19 | 0.97±0.003 $^{c}$ | 2.66 | 0.95±0.002 $^{e}$ | 2.47 | 1±0.001 $^{a}$ | 1.00 | 0.98±0.021 | 2.13 |
| TD | 1.84±0.020 $^{d}$ | 11.90 | 1.72±0.011$^{f}$ | 6.88 | 2.10±0.019$^{c}$ | 11.72 | 1.74±0.011$^{ef}$ | 7.90 | 1.59±0.012$^{g}$ | 8.65 | 1.80±0.014 $^{de}$ | 8.12 | 2.43±0.028$^{a}$ | 11.15 | 1.76±0.021$^{ef}$ | 11.94 | 2.35±0.025$^{b}$ | 10.91 | 1.73±0.012$^{f}$ | 8.42 | 1.89±0.194 | 10.27 |

$^{abcdefgh}$ LSmeans with different letters in rows are significantly different at P≤0.001; SNK's multiple mean comparison test;
MPZ, Mekrou-Pendjari zone; CAZ, Chaîne Atacora zone; BNZ, Borgou-Nord zone; BSZ, Borgou-Sud zone; BZ, Bassila zone; CZ, Coastal zone; PoZ, Pobe zone; PlZ, Plateau zone; VOZ, Oueme Valley zone; ZZ, Zou zone; MI, Mass index; IGS, Slenderness index; IAT, Auricular index; USI, Sternum index; BI, Boniness index; IP, Pelvic index; CDI, Chest depth index; SI, Size index; Ba, Balance; IBR, Body index; PI, Pectoral index; IC, Cephalic index; BR, Body ratio; TD, Thoracic development.
