# Supplementary material for: Morphological variability within the indigenous sheep population of Benin
Source: PLoS One. 2021 Oct 19;16(10):e0258761. doi: 10.1371/journal.pone.0258761 (PMC8525752; doi:10.1371/journal.pone.0258761)
Supplement: S3 Table — (PDF) [file pone.0258761.s009.pdf]

S3 Table. Incidence of phytogeographic zones and number of parity on the type of parity

Variables	Class level	Mean (%) of observation							
		Single		Twin		Triplet/Quadruplet		Multiple	
		LSmean	SE	LSmean	SE	LSmean	SE	LSmean	SE
Zones	BSZ	75[a]	3	24[cd]	3	0[c]	0	25[c]	3
	BZ	76[a]	2	24[cd]	2	0[c]	0	24[c]	2
	BNZ	76[a]	3	23[cd]	3	1[c]	0	24[c]	3
	CAZ	82[a]	2	17[d]	2	1[c]	1	18[c]	2
	MPZ	81[a]	2	19[d]	2	0[c]	0	19[c]	2
	PlZ	70[ab]	3	27[bcd]	3	3[bc]	1	30[bc]	3
	PoZ	58[c]	4	36[ab]	3	6[a]	2	42[a]	4
	VOZ	59[c]	3	37[a]	3	4[b]	1	41[a]	3
	CZ	78[a]	3	21d	3	1[c]	0	22[c]	3
	ZZ	64[bc]	3	33[abc]	3	2[bc]	1	36[ab]	3
Number of ewes parities	2	83[a]	1	17[c]	1	0[b]	0	17[c]	1
	3–5	67[b]	1	31[b]	1	2[b]	0	33[b]	1
	6 and more	50[c]	4	46[a]	4	4[a]	1	50[a]	4

For each variable, LSmeans within column with different letter (abc) are different (P<0.05).

MPZ, Mekrou-Pendjari zone; CAZ, Chaîne Atacora zone; BNZ, Borgou-Nord zone; BSZ, Borgou-Sud zone; BZ, Bassila zone; CZ, Coastal zone; PoZ, Pobe zone; PlZ, Plateau zone; VOZ, Oueme Valley zone; ZZ, Zou zone.
