# Supplementary material for: Morphological variability within the indigenous sheep population of Benin
Source: PLoS One. 2021 Oct 19;16(10):e0258761. doi: 10.1371/journal.pone.0258761 (PMC8525752; doi:10.1371/journal.pone.0258761)
Supplement: Attachment — Submitted filename: Response to Reviewers.docx [file pone.0258761.s011.docx]

To

The Editor of PLOS ONE

Dear Editor

We are grateful to the editors and reviewers for their time and constructive comments on our manuscript and for giving us the opportunity to publish in PLOS ONE.

Each comment has been carefully considered and responded, and we are pleased to submit hereby a revised version of the manuscript for further consideration in your esteemed journal. Changes in the initial version of the manuscript are either highlighted for added sentences or strikethrough for deleted sentences in the “Revised Manuscript with Track Changes”. We also provide below in the “Responses to Reviewers” a point-by-point response explaining how we have addressed each of the reviewers’ comments. Reviewers’ comments are in blue font and our response in black font.

Further, while revising the manuscript, following additional requirements were considered:

1- Compliance with PLOS ONE’s style.
2- Standardization of the contents to enable all readers to better understand the objective of the study, the methodological approach and the results obtained.
3- Grammar check and language correction

We hope that the revised manuscript meets the expectations of the journal and of the reviewers and look forward to the outcome of your assessment.

Yours sincerely,

On behalf of all authors,

Luc Hippolyte Dossa

Responses to comments

Editor Comments

I would also say it that you could rework the title. (skipping "reared"and "West Africa" I think), perhaps??

- Title has been changed as follows: "Morphological variability within the indigenous sheep population of Benin)"

additional requirements

1. Care has been taken to ensure that our manuscript meets PLOS ONE's style requirements.
2. Fig 1: Another map has been produced and the authors have been quoted as recommended.

Reviewer #1 comments

- The introduction need to be larger, we need to get more information about the importance of this adaptive population, the importance of the study of molecular characterization to future selection scheme

- The introduction has been revised accordingly, as follows:

Lines 53-93: “It is commonly accepted that two main types of sheep, the Djallonké (S1 and S2 Figs) and the Sahelian (S3 and S4 Figs), are encountered throughout the country [10,11,12]. Djallonké, also named West African Dwarf sheep, seemed to originate from the type of fine-tailed and hairy sheep native to Western Asia, having migrated to Africa through the Isthmus of Suez and Bab el Mandeb, and was the only type of sheep type on the continent until the third millennium BC. [13]. Widely distributed in West Africa, the Djallonké sheep is mainly raised for meat [10,11,14]. It is particularly adapted to coastal areas [15] because of its resistance to trypanosomiasis [12,16]. However, it would have undergone significant phenotypic changes over time [9,12]. Generally, Djallonké sheep are assimilated to small-sized animals with straight facial profile, small narrow erected ears and a hairy short coat [14,19]. Contrarily to ewes, rams are horned and have a heavy white or pied mane with black forequarters and white hindquarters. Existence in this sheep type of two sub-types differentiated by size has been distinguished [18,19]: the larger in the Sudanian zone and the smaller in the Guinean zone further south [9,17].

Sahelian sheep regroup all long legged sheep breeds known under different ethnical and local names in the semi-arid and arid zones of West-African Sahel [9]. Like Djallonké sheep, Sahelian sheep are thought to be descended from the fine-tailed and hairy sheep [9]. Although notorious for not surviving in humid areas [20], they are increasingly encountered in different humid localities of Benin for recent years [11,12], reflecting their progressive adaptation to less dry climates. In the Sahelian pastoral and agro-pastoral production systems, they are used for meat and milk production [14]. Sahelian sheep have concave facial profile, pendulous long ears, a long thin tail and diverse coat colour [9]. A typical characteristic of Sahelian ram is the absence of mane. As in several West African countries, many crossbreeds between Sahelian and Djallonké sheep (S5 and S6 Figs) are present in Benin with various intermediate body sizes.

The lack of consistent knowledge on the genetic diversity of West African sheep populations, as well as on their specific traits constitutes the major constraint for the implementation of sound programs towards their genetic improvement and sustainable use. Moreover, the presence of unknown sub-groups within each of these two known groups of sheep, as well as the occurrence of crossbreeding can lead to certain ambiguities when it comes to distinguish certain individuals according to well-defined breed standards. According to FAO (2012) [21], phenotypic and molecular characterizations are important tools for breed documentation, a first step towards the development of strategies for their sustainable use, management and conservation. To date, neither of these two characterization tools have not been covered in depth for the Beninese sheep population. Hence, in order to further document the existing diversity and to explore the spatial distribution within the indigenous sheep population of Benin, the morphological characterization based on a large panel of collected morphology/phenotypic traits was considered here as a primary study. We hypothesized that the sheep population of Benin is greatly diverse and unevenly distributed according to ecological conditions.

The current study aims at establishing the relationships among sheep morphometric traits and the ten phytogeographical zones of Benin using univariate analyses and then, explore the presence of sheep subpopulations in the Beninese indigenous sheep population using multivariate analyses. The findings of this study will provide the basis for a sound molecular study on the same samples, based on both Simple Sequence Repeat (SSR) and Single Nucleotide Polymorphic (SNP) markers genotyping. Morphological data could then be compared to molecular data and association analyses (i.e. Genome-wide association studies) performed to appropriately address possible breeding strategies for the indigenous sheep population of Benin.”

- You should have more information about the animal material, the management, the performances

- More information about these recommendations was included in the introduction, as indicated above.

- In the result part, it will be better to add the CPA analysis to see more the variability according to the fixed effects used

**>** The Canonical discriminant analysis (CANDISC) is a dimension reduction technique which included CPA analysis. This multivariate statistical technique helps to identify differences among groups of individuals and improve understanding the relationships among the variables measured within those groups. More information on this procedure could be funded in “Cruz-Castillo et al 1994.

- The conclusion should be revised again

**>** The conclusion has been revised, as follows:

Lines 418-430: “The aim of this study was to explore the morphological variability of indigenous sheep reared in different phytogeographical zones of Benin. The results showed significant variations in phenotypic traits, both qualitative and quantitative among phytogeographical zones. Four sheep sub-populations were distinguished. Animals in the phytogeographical zones of Southern Benin could be assimilated to short-legged or WAD sheep, whereas those from the zones located in the northern regions of the country were much closer to the long-legged Sahelian sheep type. The intermediate sub-populations appeared to include an ecotype of WAD sheep and various crossbreeds. These results could be due to several factors like animal’s adaptation or natural selection, changes in farmers’ breeding practices, and gene flow. However, it appears that the indigenous sheep population of Benin has been subjected to very little selective breeding. Further research is ongoing to better understand the genetic, environmental and socio-economic determinants of the recorded morphological variation. A breeding program could therefore be developed and implemented for a better management of the diversity existing between and within recorded sheep sub-populations and for a sustainable production of this livestock species in Benin.”

- we needs a grammatical revision to all the paper

> All the paper has been grammatically revised

Reviewer #2 comments

General comments

In this manuscript, body measurements, indices based on those, and qualitative traits such as color and ear type were studied for 1240 adult ewes from different areas in Benin. Four types/subpopulations of animals were detected and similarities to sheep in nearby countries were discussed. The study is motivated by the need for knowledge about genetic diversity. In the abstract, it is mentioned that it is a prelude to molecular (genetic?) characterization, but this is not mentioned again in the rest of the manuscript. It would be good to clarify if that is the intention for future studies.

- This has been clarified in the introduction, as follows:

L 78-93: “According to FAO (2012) [21], phenotypic and molecular characterizations are important tools for breed documentation, a first step towards the development of strategies for their sustainable use, management and conservation. To date, neither of these two characterization tools have not been covered in depth for the Beninese sheep population. Hence, in order to further document the existing diversity and to explore the spatial distribution within the indigenous sheep population of Benin, the morphological characterization based on a large panel of collected morphology/phenotypic traits was considered here as a primary study. We hypothesized that the sheep population of Benin is greatly diverse and unevenly distributed according to ecological conditions.

The current study aims at establishing the relationships among sheep morphometric traits and the ten phytogeographical zones of Benin using univariate analyses and then, explore the presence of sheep subpopulations in the Beninese indigenous sheep population using multivariate analyses. The findings of this study will provide the basis for a sound molecular study on the same samples, based on both Simple Sequence Repeat (SSR) and Single Nucleotide Polymorphic (SNP) markers genotyping. Morphological data could then be compared to molecular data and association analyses (i.e. Genome-wide association studies) performed to appropriately address possible breeding strategies for the indigenous sheep population of Benin.”

The manuscript is generally well written, and it is important to characterize the local genetic diversity of indigenous domestic animal populations, which seems not to be done previously for sheep in Benin.

The large number of traits and areas (and abbreviations!) mentioned makes it very important to be clear and consistent in the description and this could be improved to some degree, especially in the tables that need a bit more explanations.

- This has been improved through the clarification of some trait definitions in the Table 2. Moreover, a graphical illustration of all the morphometric measurements used in the study is now suggested as a new figure (FIG 2).

To a foreign reader, it is not very clear if there are any defined sheep breeds in the country at all, for example "exotic" breeds, in addition to the indigenous? What type of production are the sheep used for? Mainly meat production or also e.g. milk and does this differ between the areas studied?

The discussion could be made a bit more interesting. In the discussion there is some mentioning of different sheep types in different areas due to evolutionary adaptions, but this is not well covered. Are there e.g. different climatic zones that make different ear or fur color types more beneficial for survival, or are such trait differences mainly a result of what humans favored for other reasons? The same with e.g. body size or type, is there for example a logical connection to smaller body size in areas with harsher conditions and less feed/grass supply, or has it more to do with what the sheep have been used/selected for (such as meat production) etc.?

Information about breeds/types have been added as follows:

Lines 53-93: “It is commonly accepted that two main types of sheep, the Djallonké (S1 and S2 Figs) and the Sahelian (S3 and S4 Figs), are encountered throughout the country [10,11,12]. Djallonké, also named West African Dwarf sheep, seemed to originate from the type of fine-tailed and hairy sheep native to Western Asia, having migrated to Africa through the Isthmus of Suez and Bab el Mandeb, and was the only type of sheep type on the continent until the third millennium BC. [13]. Widely distributed in West Africa, the Djallonké sheep is mainly raised for meat [10,11,14]. It is particularly adapted to coastal areas [15] because of its resistance to trypanosomiasis [12,16]. However, it would have undergone significant phenotypic changes over time [9,12]. Generally, Djallonké sheep are assimilated to small-sized animals with straight facial profile, small narrow erected ears and a hairy short coat [14,19]. Contrarily to ewes, rams are horned and have a heavy white or pied mane with black forequarters and white hindquarters. Existence in this sheep type of two sub-types differentiated by size has been distinguished [18,19]: the larger in the Sudanian zone and the smaller in the Guinean zone further south [9,17].

Sahelian sheep regroup all long legged sheep breeds known under different ethnical and local names in the semi-arid and arid zones of West-African Sahel [9]. Like Djallonké sheep, Sahelian sheep are thought to be descended from the fine-tailed and hairy sheep [9]. Although notorious for not surviving in humid areas [20], they are increasingly encountered in different humid localities of Benin for recent years [11,12], reflecting their progressive adaptation to less dry climates. In the Sahelian pastoral and agro-pastoral production systems, they are used for meat and milk production [14]. Sahelian sheep have concave facial profile, pendulous long ears, a long thin tail and diverse coat colour [9]. A typical characteristic of Sahelian ram is the absence of mane. As in several West African countries, many crossbreeds between Sahelian and Djallonké sheep (S5 and S6 Figs) are present in Benin with various intermediate body sizes.

The lack of consistent knowledge on the genetic diversity of West African sheep populations, as well as on their specific traits constitutes the major constraint for the implementation of sound programs towards their genetic improvement and sustainable use. Moreover, the presence of unknown sub-groups within each of these two known groups of sheep, as well as the occurrence of crossbreeding can lead to certain ambiguities when it comes to distinguish certain individuals according to well-defined breed standards. According to FAO (2012) [21], phenotypic and molecular characterizations are important tools for breed documentation, a first step towards the development of strategies for their sustainable use, management and conservation. To date, neither of these two characterization tools have not been covered in depth for the Beninese sheep population. Hence, in order to further document the existing diversity and to explore the spatial distribution within the indigenous sheep population of Benin, the morphological characterization based on a large panel of collected morphology/phenotypic traits was considered here as a primary study. We hypothesized that the sheep population of Benin is greatly diverse and unevenly distributed according to ecological conditions.

The current study aims at establishing the relationships among sheep morphometric traits and the ten phytogeographical zones of Benin using univariate analyses and then, explore the presence of sheep subpopulations in the Beninese indigenous sheep population using multivariate analyses. The findings of this study will provide the basis for a sound molecular study on the same samples, based on both Simple Sequence Repeat (SSR) and Single Nucleotide Polymorphic (SNP) markers genotyping. Morphological data could then be compared to molecular data and association analyses (i.e. Genome-wide association studies) performed to appropriately address possible breeding strategies for the indigenous sheep population of Benin.”

- Discussion

Lines 383-416: “This study highlights a highly diverse Beninese sheep population, within which the distribution of individuals is affected by natural and also anthropic factors. The most important natural factors at the origin of the recorded sheep diversity across the ten investigated phytogeographical zones are climate-related (temperature, humidity and/or vegetation cover) which affect the availability of feed resources and induce natural selection pressures. The anthropic factors mainly concern animal management practices in the different zones, cultural preferences and livestock marketing systems. Thus, the phenotypic traits (small size, stocky appearance, small ears, long hair), characteristics of the Djallonké sheep type that predominates in Southern Benin are likely a response to natural selection over several generations under the influence of the constraints of the environment in which the animals are raised. Furthermore, the larger phenotypic traits of the Djallonké ecotype in the PoZ, CZ and BZ could be explained, in addition to the influence of the environment, by changes in sheep farmers’ breeding practices in these phytogeographical zones, in particular the practice of crossbreeding short-legged with Long legged animals from the North. This is undoubtedly influenced by the annual flow of Sahelian animals to these regions during the cultural ceremonies of Aid El-Kebir, when sheep sacrifice takes place in Muslim households. In addition, the breeders of these areas would try to adapt to new consumer demands, as expressed by their preference for animals that possess larger physical features than the Djallonké during ceremonies and festivals. Likewise, the Sahelian sheep, which is predominant in the MPZ in North Benin, are larger and slenderer, with varied but predominantly light coats, a short hair, a long tail, dropped and larger ears. These specific traits allow them to withstand heat stress and to adapt to the savannah vegetation of trees and shrubs, predominant in these areas [44], that would be relatively harsher for small-sized sheep. Furthermore, their long legs predispose them to travel long distances in search of pastures. The BNZ and CAZ, with their intermediate climatic gradient between the humid South and the dry North, promote, on the one hand the extension of the distribution area of the Sahelian types, and on the other hand the cross-border sheep transhumance practices that are at the origin of the admixture of subpopulations observed in these two zones. Referring to transhumance, it is worth noting that during the migratory period, and in order to meet their own subsistence needs, transhumant sheep herders often sell or exchange a few heads of animals in their herds for food and salt [36]. On the other hand, the attraction of certain sheep farmers for large animals in areas hosting transhumant-herds sometimes encourages them to herd their animals to the same grazing areas in the hope of mating their animals with those kept by the transhumant herders.

Although morphological variation is largely under genetic control [29], it is subject to the influence of the environment and management practices. Thus, the preservation of local populations that adapt to their environment is essential. This calls for the development of new management strategies for sheep farming in Benin aiming at improving farm profitability through improved animal performances while preserving the diversity within the local sheep population in order to face current and future challenges in production systems, including climate change, and respond to market demand.

Specific comments

Line 57: rewrite so that it is easier to understand which reference you mean by According to [9] without searching in the ref. list. (e.g. According to FAO [9] …).

A: this sentence has been rewritten

Line 78-79: “According to FAO (2012) [21], phenotypic and molecular characterizations……”

Table 1: Does the à mean to? Perhaps a - would be clearer.
A: The term “the” has been added

Lines 110-111: “… predominant vegetation are presented in **the** Table 1.”

Line 91-95: Was pedigree records available to select unrelated animals, or how did you determine which animals were unrelated? It appears as if no records were kept about date of birth as an inspection of teeth was made to determine the age, so were there then records about parents?

No, with a few exceptions there was no written data on the pedigree of the animals for most of the farms. (No formal birth registration) but the breeders knew their animals well, the period of birth, the origin... The sampling was therefore done on the basis of their memory, the information provided by the breeders on the pedigree of the animals and/or their origin. This information has been now added and the sentence rephrased as follows: About 4 or more unrelated animals were sampled per flock based on farmers’ knowledge of the individual animals present in their sheep flocks.

Line 100: Rewrite so that you do not just say from [14], but make it easier to read and understand, e.g. by saying from a previous study…

This sentence has been rewritten

Line 133: “… traits drawn from the FAO guidelines [24] and from a previous study [26],...”

Table 2: Some descriptions of conformation measures are unclear, for example the meaning of head medium in the description of SIL, and extremities of eyes (HW), and measure a few above… for MD (a few what?), and in NL from beginning of the throat at its middle? Please go through the table and clarify, and one or several pictures illustrating the different measures would be very helpful.

- The definitions of SIL, BL, HW, MD and NL have been reviewed.
- new figure has been added to better describe the different morphometric measurements taken (Fig 2).

Line 122: Should it be ..the highest discriminating…?
This sentence has been rewritten as follows:

(Line 162-163): A stepwise discriminant analysis was performed using the PROC STEPDISC to identify the **most useful** morphometric traits and morphological indices **for further discriminant analyses**

Line 159: It would be good to remind the reader about the meaning of the IGS index as well, as was done for the other mentioned indices in this part of the text.

This sentence has been rewritten as follows:

Line 201: The highest mean values of the **slenderness** (IGS)

Line 162: Do you here mean significant differences in frequencies?

Yes. This sentence has been rewritten

Line 203:” Significant **differences** in frequencies…”

Line 174: Do you mean the ..most common..?
Yes. This sentence has been rewritten

Line 216: “Irrespective of the zone, single birth-kids were the **most** common.”

Line 178: Frequency...traits in sheep populations…?

Yes. The tittle of the Table 4 has been rewritten

Line 221: “Table 4. Frequency (%) of qualitative traits **in** sheep population…”

Table 4: An explanation of the zone abbreviations is needed, and so is an explanation of what the chi and P-values are for. The color names Dominant white etc. can be a bit confusing as they are not the same as used in the text, and are often used in other articles to describe certain genes or inheritance patterns of colors. The n= in the table seem to be the same for all traits in each zone, and should then not be repeated multiple times, it could be put in the first or second row. The back profile is a bit unclear, in the text it says slopes up towards the withers (higher at withers than at rump?) and in Table 4 it says descending towards withers which sounds like the opposite?
The zone abbreviations were described, and chi^2^ and P-values were explained below the Table 4. The qualitative variable denomination, such as the color names, have been reviewed in the Table 4 as well as in the manuscript. The n= has rewritten in the first lines and repetition has been deleted in the Table 4. The definition of back profile modalities has been corrected in the Table 4 and uniformed as well in the manuscript.

*(No, the height at withers is smaller than the height at rump)

Line 185: is the PR>F needed?
Yes, it allows to specify the level of choice for the thirty-two significant variables included in the rest of the analyzes.

Table 5: an explanation of what is meant by Number in traits should be given (the number of variables in the model), and the table description could be a bit longer and more informative. Consider if all given decimals are needed, e.g. for the average squared canonical correlations?
Explanation of what is meant by Number in traits has been given below the table 5.

The table description has been improved in the Table title and further described in the manuscript: Lines 229-233: “The traits RW and SH showed higher partial R2 and F-values illustrating their greater discriminant power compared with the other variables used to assess the morphological diversity in Benin sheep population. Nevertheless, the use of the thirty-two significant (P<0.0001 for column Pr > F) quantitative variables (i.e., 22 quantitative linear body traits and 10 morphological index) in the”.

The decimal numbers have been rounded for Wilks’ lambda (λ) and average squared canonical correlations added in the Table 5.

Table 6: Also for this table it would be helpful with a bit more explanations, are the SE for the correlations, proportion of.., cumulative what?
Yes it is the approximate SE of the canonical correlations. More information has been given for these in the Table 6, as follows:

Approximate Standard Error = Approximate Standard Error of the canonical correlations, Proportion = Proportion of the eigenvalue sum

cumulative = Cumulative proportion of the eigenvalue sum

Table 8: Again, some more clear descriptions could be provided: What is rate for example - proportion of animals from one zone classified in another/wrong zone?
The description of Rate and Priors has been given below the Table 8 as follows:

Rate: proportion of misclassified observation in each phytogeographical zone

Priors: Priors probabilities of group membership

Line 250-251: How do you know these are due to evolutionary adaptation? Could not differences in size be due to different management and feeding intensity in addition to genetic adaptation? And there may have been selection for growth and for colors that the animal owners in certain area prefer?

- We have just suspected that the variations are an adaptive response. This sentence has been rewritten in Lines 312-318: “In this study, we aimed to further document the existing diversity and the spatial distribution within the sheep population raised in Benin based on a large panel of collected qualitative and quantitative traits. Univariate analyses revealed significant differences among phytogeographical zones for all measured morphological traits and derived indices, suggesting possible influence of these zones on the evolutionary adaption of the sheep population regarding these morphological traits. This result is in line with findings of N’Goran et al. (2019) [16] who reported a significant impact of the breeding area on morphological traits in the sheep population from Ivory-Coast.~~”~~
- Yes, differences in size could also be due to different management in addition to genetic adaptation and this has been added in the discussion, as follows:

Lines 382-410: “This study highlights a highly diverse Beninese sheep population, within which the distribution of individuals is affected by natural and also anthropic factors. The most important natural factors at the origin of the recorded sheep diversity across the ten investigated phytogeographical zones are climate-related (temperature, humidity and/or vegetation cover) which affect the availability of feed resources and induce natural selection pressures. The anthropic factors mainly concern animal management practices in the different zones, cultural preferences and livestock marketing systems. Thus, the phenotypic traits (small size, stocky appearance, small ears, long hair), characteristics of the Djallonké sheep type that predominates in Southern Benin are likely a response to natural selection over several generations under the influence of the constraints of the environment in which the animals are raised. Furthermore, the larger phenotypic traits of the Djallonké ecotype in the PoZ, CZ and BZ could be explained, in addition to the influence of the environment, by changes in sheep farmers’ breeding practices in these phytogeographical zones, in particular the practice of crossbreeding short-legged with Long legged animals from the North. This is undoubtedly influenced by the annual flow of Sahelian animals to these regions during the cultural ceremonies of Aid El-Kebir, when sheep sacrifice takes place in Muslim households. In addition, the breeders of these areas would try to adapt to new consumer demands, as expressed by their preference for animals that possess larger physical features than the Djallonké during ceremonies and festivals. Likewise, the Sahelian sheep, which is predominant in the MPZ in North Benin, are larger and slenderer, with varied but predominantly light coats, a short hair, a long tail, dropped and larger ears. These specific traits allow them to withstand heat stress and to adapt to the savannah vegetation of trees and shrubs, predominant in these areas [44], that would be relatively harsher for small-sized sheep. Furthermore, their long legs predispose them to travel long distances in search of pastures. The BNZ and CAZ, with their intermediate climatic gradient between the humid South and the dry North, promote, on the one hand the extension of the distribution area of the Sahelian types, and on the other hand the cross-border sheep transhumance practices that are at the origin of the admixture of subpopulations observed in these two zones. Referring to transhumance, it is worth noting that during the migratory period, and in order to meet their own subsistence needs, transhumant sheep herders often sell or exchange a few heads of animals in their herds for food and salt [36]. On the other hand, the attraction of certain sheep farmers for large animals in areas hosting transhumant-herds sometimes encourages them to herd their animals to the same grazing areas in the hope of mating their animals with those kept by the transhumant herders.”

Line 251: the trait thoracic depth has not been defined previously, only thoracic development.
This definition was an error. The right definition has been corrected in Line 318: “The mean values of thoracic development (TD), greater…”

Line 292-293: What do you mean by crossbreeding in this case, are there defined breeds that are crossed or do you mean that animals from different regions or of different types are crossbred?

We mean that in these areas farmers cross different types from the same location and from different regions. Now, this sentence has been deleted to avoid repetition in the text.

Line 297-299: do you really define crossbreeding (and natural selection) as an environmental factor (as opposed to genetic)?

No, it is an error of formulation. This sentence has been improved as follows: Lines 362-363: “This result thus confirms the influence of environmental factors on the morphology of sheep [39-42], in addition to transhumance and management practices.”
