# Supplementary material for: Morphological variability within the indigenous sheep population of Benin
Source: PLoS One. 2021 Oct 19;16(10):e0258761. doi: 10.1371/journal.pone.0258761 (PMC8525752; doi:10.1371/journal.pone.0258761)
Supplement: Attachment — Submitted filename: PLOSbeninSheep2.docx [file pone.0258761.s012.docx]

PLOS_BeninSheep2

The manuscript is greatly improved. There are however several issues remaining.

1. We asked for the results to be put into a broader focus. The background you provided is all about sheep in Benin, but this can be scaled back and a broader geographic view taken. This makes the study more general, to students of sheep on the African continent and elsewhere. What is know about radiation or adaptive evolution of sheep breeds in other countries or in other parts of Africa?
2. Improve the grammar and wording. Get an outsider to review the paper for you.

Examples of sentences that need improving

Abstract: “Good knowledge…”

Line 50 “In West Africa, sheep populations are raised under harsh and diverse ecological conditions, which may have led to the evolution of…”

Line 65 “Djallonké sheep are assimilated to small-sized”?? better verb?

Line 79 drop “consistent”

Line 85

“To date, neither of these two characterization tools have not been covered in depth for of the Beninese sheep populations.”????

There are several examples where extra words are used, that can be dropped. E.g. 510 “fully acknowledge”

3. Indicate the origin of the map, software, database etc.
4. Figure legends should be extended. They should better describe the content of the figure.
5. Tone downs statements of adaptive value of traits throughout manuscript.
   a. For instance in discussion (line 441) “These specific traits may allow them to withstand heat stress and to adapt”
