# Supplementary material for: Morphological variability within the indigenous sheep population of Benin
Source: PLoS One. 2021 Oct 19;16(10):e0258761. doi: 10.1371/journal.pone.0258761 (PMC8525752; doi:10.1371/journal.pone.0258761)
Supplement: Attachment — Submitted filename: Response to Reviewers Comments.docx [file pone.0258761.s013.docx]

**Response to Reviewers**

**Reviewers’ comments are in black font and our responses in blue font.**

Additional Editor Comments (if provided):

**PLOS_BeninSheep2**
The manuscript is greatly improved. There are however several issues remaining.

1. We asked for the results to be put into a broader focus. The background you provided is all about sheep in Benin, but this can be scaled back and a broader geographic view taken. This makes the study more general, to students of sheep on the African continent and elsewhere. What is known about radiation or adaptive evolution of sheep breeds in other countries or in other parts of Africa?

The results have been put into a broader focus as follows:

- Lines 379-382:” This study highlights a highly diverse sheep population in Benin, as in other African countries (e.g., Burkina Faso, Ivory Coast, Togo, and Nigeria), within which the distribution of individuals is affected by natural and also anthropogenic factors. Thus, the sheep subgroups observed in the different phytogeographic zones of Benin also exist in other African countries in similar or different environments [16,31,36,44].”
- Lines 413-417: “This calls for the development of new management strategies for sheep farming in Benin as well as in other African countries aiming to improve farm profitability by improving animal performance while preserving the diversity within the local sheep populations. In this way, sheep farming would overcome current and future challenges in production systems in Africa, including climate change and market demand.”

2. Improve the grammar and wording. Get an outsider to review the paper for you.
Examples of sentences that need improving
Abstract: "Good knowledge…"
Line 50 "In West Africa, sheep populations are raised under harsh and diverse ecological conditions, which may have led to the evolution of…"
Line 65 "Djallonké sheep are assimilated to small-sized"?? better verb?
Line 79 drop "consistent"
Line 85 "To date, neither of these two characterization tools have not been covered in depth for of the Beninese sheep populations."????
There are several examples where extra words are used, that can be dropped. E.g. 510 "fully acknowledge"

All the manuscript has been edited to ensure language and grammar accuracy be Editage (<https://app.editage.com>). The Certificate has been provided in the cover letter submitted to the editor. Some sentences have been improved as follows:

e.g. Line 15: “Knowledge of both the genetic diversity and…”

Lines 45-46: “In West Africa, sheep populations are raised under harsh and diverse ecological conditions, which may have led to the evolution of diversified adaptive traits for their survival [2,8].”

3. Indicate the origin of the map, software, database etc.

The origin of the map given on the Figure 1 has been corrected (Source: Topographic Map IGN, 1992. Field Work, 2018-2020. WHANNOU Habib R. V. adapted from Adomou (2005)) and the software used to draw the map has been added in the tittle of the figure as follows: “Map of the vegetation zones and phytogeographic zones of Benin showing the 32 communes sampled to assess the morphological variability within the indigenous sheep population of Benin. The map was made using QGIS 3.8 [24].”

4. Figure legends should be extended. They should better describe the content of the figure.

The legends have better described in the manuscript. E.g.: “Fig 1. Map of the vegetation zones and phytogeographic zones of Benin showing the 32 communes sampled to assess the morphological variability within the indigenous sheep population of Benin. The map was made using QGIS 3.8 [24].”

5. Tone downs statements of adaptive value of traits throughout manuscript.
a. For instance, in discussion (line 441) "These specific traits may allow them to withstand heat stress and to adapt"

The statements of adaptive value traits have been tone down through the manuscript. E.g. Lines 399-402:” These specific traits allow them to reflect solar radiation better, and thus, are less prone to heat stress [45]. In addition, their long legs predispose them to travel long distances when searching for pastures. Moreover, their large height allows them to feed easily in tree and shrubs savannah pastures, which are predominant in these regions [22,23].”

**#Journal Requirements:**

Please review your reference list to ensure that it is complete and correct. If you have cited papers that have been retracted, please include the rationale for doing so in the manuscript text, or remove these references and replace them with relevant current references. Any changes to the reference list should be mentioned in the rebuttal letter that accompanies your revised manuscript. If you need to cite a retracted article, indicate the article's retracted status in the References list and also include a citation and full reference for the retraction notice.

The reference list has been reviewed and some changes have been done. Citation errors have been corrected in the lines 61-64: “small narrow-erected ears, and a hairy short coat [14,17]. In contrast to ewes, rams are horned and have a heavy white or pied mane black forequarters and white hindquarters. Two sub-breeds of Djallonké sheep have been identified based on size [18,19]: the larger breed is found in the Sudanian zone, and the smaller breed in the Guinean zone further south [9,18].”

A new reference has been cited in the tittle of Figure 1 as follows: “The map was made using QGIS 3.8 [24].”

**#Specific comments**

Line 28: do you mean among (or between) phytogeographical zones?

This word has been corrected in the text in Line 26-27: “Univariate analyses indicated that all quantitative linear body measurements varied significantly (P<0.05) **across** the phytogeographic zones”

Line 29 + 31: I assume that with the precision of measurements in cm that was possible, it would be enough to give the mean with one decimal here, and the SE (is it SE - please clarify) with two decimals. (Also on line 200-201).

For consistence, the mean values have been given with one decimal and the SE with two decimals throughout the manuscript.

Line 55: seems instead of seemed?

This word has been corrected in the text in Line 54: “, also named West African Dwarf sheep, seems to originate....”

Line 59: change 'would have' to something else like 'is likely to have'

This word group has been changed in the sentence in Line 59: “However, Djallonké sheep may have undergone …”

Line 60: could 'assimilated to' be removed here?

This word group has been removed in Line 60: “Generally, Djallonké/WAD sheep are small-sized animals”

Line 62: change 'with black forequarters' to ', black forequarters'.

This word group has been changed in Lines 61-62 as follows: “rams are horned and have a heavy white or pied mane black forequarters and white hindquarters. …”

Line 63: rewrite e.g. as 'Two sub-types of ……. differentiated by size have been distinguished …'

This sentence has been changed in Lines 62-63: “Two sub-breeds of Djallonké sheep have been identified based on size [18,19] ….”

Line 65: Regroup sounds a bit odd here to me, do you mean include or something else (e.g. comprise)?

This verb has been changed in Line 65: “Sahelian sheep include all long-legged sheep breeds ….”

Line 70: Some of the pictures give the impression that the noses tend to be convex rather than concave? Please check this and make sure to change throughout the manuscript (including tables and figure texts) IF you would find out that you wrote this wrong.

Yes, it was a mistake. This word has been corrected throughout the manuscript.

Line 101: Consider changing 'into' to 'in'.

This word has been corrected in the line 108:” This study was conducted in the 10 phytogeographic zones ….”

Line 125: Rewrite 'Thus, a total of 1240 ewes that were at least two years old and multiparous (at least two lambings) ....'. I think farrowing is more commonly used for pigs (?).

This sentence has been rewritten and the words “farrowing” and “kids” has been corrected in the lines 127-128 as follows: “Thus, a total of 1240 ewes that were at least two years old and multiparous (at least two lambings) were randomly selected, described and phenotypically characterized.”

Table 3: perhaps references would be a better word than authors on the third column head?

The word “Authors” has been corrected in the table 3.

Line 216-217: Wouldn't lamb be better to use than kid here (e.g. single-born lamb)

This word has been corrected in the lines 213-215: “Irrespective of the zone, single-born lambs were the most common. The highest percentages of twin-born lambs were recorded in the Oueme Valley, Pobe, and Zou zones, whereas the highest proportions of triplets and quadruplets were recorded in the Pobe zone. The percentage of multiple births appeared to ….”

Line 229: Clarify - significant for what? For example, significantly contributing to discrimination between groups (?).

This has been clarified in lines 225-226 as follows:” … (i.e., 25 quantitative linear body traits and 14 morphological indices) included in the analysis significantly contribute to discrimination between the phytogeographic zones (P<0.0001).”

Table 7: It would be easier to read if you use the same number of decimals (e.g. 2) for all variables in the table, and adjust it so that the dots are beneath each other.

This has been corrected in the table and the values has been adjusted to have dots beneath each other.

Table 8: Please explain more clearly what is above vs below the diagonal in the table.

The values above vs below the diagonal in the table 8 has been explained below the table as follows: “Values above and/or below the diagonal represent the percentage of individuals from other phytogeographic zones present in the zone considered by the diagonal value.”

Line 335: what is the color pie-brown, is that not also a bicolor/piebald?

Yes, it is also bicolor coat.
Line 347 (and 335): are the Oudah and Bali-bali the same or different breeds/types?

- No, Oudah and Bali-bali are Sahelian sheep but they are two different breeds.

Line 355-356: One decimal would be enough here I think, also for the means in Line 371-372.

For consistence with the others part of the manuscript, the mean values have been given with one decimal and the SE with two decimals.

Line 421: is assimilated the correct word here?

This verb has been changed in line 421: “Four sheep sub-populations were identified. Animals in the phytogeographic zones of Southern Benin could be identified as short-legged...”
