# Supplementary material for: Morphological variability within the indigenous sheep population of Benin
Source: PLoS One. 2021 Oct 19;16(10):e0258761. doi: 10.1371/journal.pone.0258761 (PMC8525752; doi:10.1371/journal.pone.0258761)
Supplement: Attachment — Submitted filename: Response to Reviewers.docx [file pone.0258761.s014.docx]

**Response to Reviewers**

Additional Editor Comments (if provided):

**Comment 1**:

The manuscript is greatly improved.

There are two minor issues, that should only take a day to fix.

In the previous edit we requested you … [ down statements of adaptive value of traits throughout manuscript.]. this was not adhered to.

Line 452 "These specific traits allow them to reflect solar radiation better, and thus, are less prone to heat stress [45]. In addition, their long legs predispose them to travel long distances when searching for pastures. Moreover, their large height allows them to feed easily in tree and shrubs savannah pastures, which are predominant in these regions [22,23]".

Please rewrite this, remove the assertive tone and make this more nuanced. Please flank with caveats like "Several hypothesis about the adaptive value of these traits have been put forth. …. But confirmation of these hypotheses requires further study and remains inconclusive".

**Response**: We sincerely thank the editor for his positive feedback on our revised submission.

The statements of adaptive value traits have revised as suggested throughout the manuscript as follows:

- Lines 358-360: “This result **is likely to** confirm the effect of environmental factors on the morphology of sheep [40-43] and transhumance and management practices.”
- Lines 382-385: “The most important natural factors at the origin of the recorded sheep diversity across the 10 investigated phytogeographic zones **might be** climate-related factors (temperature, humidity, and/or vegetation cover), which affect the availability of feed resources and induce natural selection pressures.”
- Lines 399-404: “**Several hypotheses about the adaptive value of these traits have been put forth. For instance, Gaughan et al. (2018) [45] argue** that these specific traits **might** allow them to reflect solar radiation better, and thus, **to be** less prone to heat stress. In addition, **according to these authors** their long legs **might** predispose them to travel long distances when searching for pastures. Moreover, their large height **might** allow them to feed easily in tree and shrubs savannah pastures, which are predominant in these regions [22,23]. **But confirmation of these hypotheses requires further study and remains inconclusive.**”

**Comment 2**:

Line 484. Is there a citation for this conclusion? "However, it appears that the indigenous sheep population of Benin has been subjected to very little selective breeding." It is not a natural conclusion from your data.

Suggest you remove this if no citation is available.

**Response**: This sentence has been removed

**#Journal Requirements:**

Please review your reference list to ensure that it is complete and correct. If you have cited papers that have been retracted, please include the rationale for doing so in the manuscript text, or remove these references and replace them with relevant current references. Any changes to the reference list should be mentioned in the rebuttal letter that accompanies your revised manuscript. If you need to cite a retracted article, indicate the article's retracted status in the References list and also include a citation and full reference for the retraction notice.

**Response**: The reference list has been reviewed and some changes made from line 463 to line 591 to respond with Compliance with PLOS ONE’s style.

Two new references [**46,47**] have been cited on line 415 and added to the list
